# Aging Effects on Epicardial Adipose Tissue

**DOI:** 10.3389/fragi.2021.666260

**Published:** 2021-05-13

**Authors:** Gianluca Iacobellis

**Affiliations:** Division of Diabetes, Endocrinology and Metabolism, Department of Medicine, Miller School of Medicine, University of Miami, Miami, FL, United States

**Keywords:** epicardial, epicardial fat, Brown fat, aging, coronary arteiy disease

## Abstract

Epicardial fat is the visceral fat of the heart. Epicardial fat is a white adipose tissue, but it displays also brown-fat like or beige fat features. Under physiological conditions, epicardial fat has cardioprotective functions such as free fatty acids supply and thermoregulation of the adjacent myocardium. Epicardial adipose tissue encounters changes in the transition from embryological to childhood and then to adult life. Aging can affect the function and morphology of epicardial fat, more likely in women than in men. The effect of aging on the brown fat properties of the epicardial fat is the most prominent and with the greatest clinical implications. It is promising to know that epicardial fat responds to newer pharmaceutical drugs modulating the adipose tissue and potentially restoring its browning effects. Epicardial fat pro-inflammatory secretome is down-regulated in end-stage coronary artery disease. Chronic ischemia and adverse hemodynamic conditions can also affect the regulatory component of the epicardial fat. Epicardial fat may incur in apoptotic and fibrotic changes that alter its transcriptome and proteasome. In conclusion, aging and advanced stage of chronic diseases are likely to influence and affect epicardial fat genes and function**.** Whether the downregulation of the epicardial fat tissue is due more to aging than advancing stages of coronary artery disease, or more likely to the combination of both, would be object of future investigations.

## Epicardial Fat

Epicardial fat lies between the myocardium and the visceral layer of the epicardium (Iacobellis et al., 2005a; Iacobellis and Bianco, 2011; Iacobellis, 2015). Embriologically, epicardial fat evolves from brown adipose tissue (Marchington et al., 1989; Marchington and Pond, 1990). Epicardial fat is a white adipose tissue, but it displays also brown-fat like or beige fat features. Microscopically, epicardial fat is composed of adipocytes, nerve tissues, vascular and immune cells (Iacobellis and Bianco, 2011). There is no anatomical barrier between the fat and myocardium. This is one of the most unique features of the epicardial fat as it allows a cross-talk of the fat with the adjacent myocardium. The physiological function of the epicardial fat is to supply the myocardium with energy and thermoregulation. Epicardial fat can also secrete adipokines with cardioprotective effects (Iacobellis et al., 2005a; Iacobellis and Bianco, 2011; Iacobellis, 2015). Nevertheless, under pathological circumstances, epicardial adipocytes may lose this cardioprotective function and turn into pro inflammatory cells. Aging and advanced stage of chronic diseases can influence and affect epicardial fat genetic profile and function, although little is known about this effect.

## Cardioprotective Role of Epicardial Fat

Epicardial fat plays a key role in the myocardial energy homeostasis providing the heart with free fatty acids and heat. Epicardial fat has the greatest capacity for free fatty acids release and uptake, among any other visceral fat depots (Marchington et al., 1989; Marchington and Pond, 1990). Physiologically, epicardial fat serves as a buffer against the lipotoxic effect of high fatty acids levels. Epicardial fat incorporates fatty acids as energy store that can be used as fuel for the myocardium. Due to the vicinity to the heart, free fatty acids are transported from the epicardial fat directly into the myocardium. Epicardial fat also expresses fatty-acid-binding protein four that regulates coronary arterial tone and help the free fatty acids influx (Vural et al., 2008; Pezeshkian and Mahtabipour, 2013).

Epicardial adipose tissue is a metabolically active endocrine/paracrine organ (Iacobellis and Barbaro, 2008). It can protect and feed the myocardium throughout the secretion of anti-inflammatory cytokines and regulatory factors (Iacobellis and Barbaro, 2019). Epicardial fat expresses genes encoding for cardio-protective cytokines such as adiponectin and adrenomedullin that can reach out directly to the myocardium and coronary arteries, through either paracrine or vasocrine mechanisms (Iacobellis et al., 2005b; Judkin et al., 2005; Iacobellis et al., 2009; Iacobellis et al., 2009; Iacobellis et al., 2010). Adiponectin is expressed in the epicardial adipose tissue, as first discovered by Iacobellis et al. (2005b). Epicardial fat adiponectin can reduce local inflammation and lipid accumulation in the macrophages. Intracoronary plasma adiponectin levels are related to epicardial fat adiponectin protein expression in patients with coronary artery disease (Iacobellis et al., 2009). Adrenomedullin can also have protective effects to the heart (Silaghi et al., 2007). Adrenomedullin receptors are expressed in the epicardial fat and the adipokine is secreted by the epicardial adipocytes and macrophages (Silaghi et al., 2007). However, the regulation of both expression and secretion of these cardioprotective adipokines is still unknown.

## Epicardial as Brown Fat

The role of brown fat in human beings is a topic of growing interest, far to be clearly understood. Brown adipose tissue produces heat and nonshivering thermogenesis, in response to cold temperatures. Brown fat produces energy through uncoupling protein (UCP)-1-mediated heat production. Activation of brown fat can increase energy expenditure, reduce adiposity, and potentially help with weight loss (Sacks and Fain, 2007). Unfortunately, the majority of the brown adipocytes are lost in the transition from infancy to adult life in humans.

Human epicardial fat has brown fat features and properties (Sacks and Fain, 2007; Sacks et al., 2009; Sacks et al., 2013). Histologically, epicardial fat should better defined as beige fat, given the histological resemblance with *in vitro* in beige lineage adipocytes (Sacks et al., 2013). Small unilocular adipocytes without UCP-1 immuno-staining have been identified in epicardial fat. Brown fat specific gene UCP-1 and other brown fat regulatory genes, are all highly expressed in human epicardial fat (Sacks et al., 2009). UCP-1 is higher in human epicardial fat in comparison to other fat depots, and undetectable in subcutaneous fat. Epicardial fat can provide direct heating to the myocardium and protect the heart during poor hemodynamic conditions, such ischemia or hypoxia. However, the role and potential of epicardial fat to act as brown fat is still under investigation.

## Aging Effects on Epicardial Brown-Fat Activity

Aging can influence the function of epicardial fat, more likely in women than in men. Epicardial fat secretome was decreased in older female rats (Fei et al., 2010). Epicardial fat adiponectin and interleukin -6 genes were downregulated in the aged and very aged rats (Fei et al., 2010). Interestingly, macrophage markers decreased with aging, in both female and male rats. The transcriptome of epicardial fat obesity-related genes decreased with aging in female, but not in male rats (Kocher et al., 2017). Adipocyte remodeling and function could be under the influence of the sexual hormonal control. The loss of epicardial fat function with aging seems to be more prominent in female than in male rats. The effect of aging on epicardial fat has been not fully evaluated in humans.

The potential thermogenic properties of epicardial fat could be the first to be lost with aging. It would be interesting to see if the aging effects on epicardial fat could contribute to the higher cardiovascular risk in post-menopausal women.

Epicardial fat brown fat-like activity and function can also decrease with aging (Ojha et al., 2016). Remarkably, the morphology of epicardial fat adipocytes significantly differs among the neonate, infant, and child. The structural cellular changes can affect the secretome, including UCP1 secretion. Epicardial fat maintains a significant number of UCP1-positive cells into childhood. Autoptic studies revealed evidence of brown fat in the pericardium and epicardial fat in human infants and young children (Fainberg et al., 2018). The proportion of brown adipocytes decreases in favor of more unilocular white adipocytes in older subjects. These changes suggest the transition from brown to beige fat of epicardial adipose tissue in adult life. The loss of epicardial fat brown adipocytes with age and maturation was analyzed in young sheep during the first month of life (Fainberg et al., 2018). UCP1 signal was reduced by 28 days of age, although UCP1 was still present in the epicardial fat as compared to the other fat depots (Fainberg et al., 2018).

The adaption of epicardial fat to pathological conditions is still unclear. Upregulation may occur as compensatory response to the chronic hypoxic insult whereas down-regulation would be expected as consequence of the fibrotic and apoptotic involution in the end-state organ diseases (McAninch et al., 2015). Nevertheless results are controversial and not yet conclusive. Alternatively, other possible cardioprotective pathway genes involving peroxisome proliferator-activated receptor-gamma coactivator alpha (PGC-1α), involved in adipocyte browning and thermogenic activation, have been suggested (Singh et al., 2019). Epicardial fat heme oxygenase-1 (HO1) PGC-1α may modulate inflammation, mitochondrial activity and left ventricle function (Singh et al., 2019). Epicardial fat PGC-1α and HO-1 can therefore play a role in the mitochondrial function and biogenesis. A reduction of HO-1 PGC-1α and PR domain containing (Cypess et al., 2009) (PRDM16), a brown adipocyte differentiation transcription factor, in epicardial fat compared to visceral controls was observed in cardiomyopathies (Singh et al., 2019). Notably, up-regulation of brown fat and mitochondrial signaling genes was associated with a decrease in epicardial fat inflammation.

If epicardial fat turns into a beige fat depot in adult life, it can keep the genetic potential to switch back on its early brown fat activity. In fact, UCP-1 expression, was present and abundant in epicardial of adult humans. Aging and advanced coronary artery disease may affect the potential epicardial brown fat properties.

## Epicardial Fat Downregulation in Aged Coronary Artery Disease

Epicardial adipose tissue is enriched in genes encoding for regulatory factors such as genes coding for potassium channel activity, mesoderm development, wound healing, nuclear signaling pathways, protein catabolism and innate immunity pathway (McAninch et al., 2015). Nevertheless, most of these genes are down-regulated in the epicardial fat of patients with coronary artery disease.

Epicardial fat expresses and secretes adipokines with potential protective effects to the adjacent myocardium. The expression of these cardioprotective adipokines, such as adiponectin and adrenomedullin, is reduced in advanced coronary artery disease (McAninch et al., 2015). Epicardial fat adiponectin protein expression is downregulated in patients with coronary artery disease when comparted to those without cardiac ischemic disease (Iacobellis et al., 2005b). The lower epicardial fat adiponectin expression can therefore contribute to favor free fatty acids myocardial accumulation over combustion.

Chronic ischemia can also downregulate epicardial fat adrenomedullin secretion in advanced stages of coronary artery disease, although cytokines levels are likely regulated by a complexity of hemodynamic factors.

Epicardial fat transcriptome is primarily characterized by markers of inflammation (Mazurek et al., 2003). However this highly inflammatory profile may paradoxically go into a sort of cellular inactivity in subjects with advanced coronary artery disease. Maladaptive mechanisms might result in depletion of the epicardial fat cellular activities as coronary artery disease progresses and worsens (Regan et al., 2003). Fibrotic and apoptotic changes within the epicardial fat are likely to occur in advanced stages of atherosclerosis. The chronic hypoxia can eventually affect the regulatory component of epicardial fat proteasome.

## Effects of Menopause on Epicardial Fat

As noted before, epicardial fat reduction of the brown fat features seems to be more prominent in female rats. How and whether this can be translated in humans is unknown. Menopause is well-known to be related to higher risk of major cardiometabolic disease (Mazurek et al., 2017). Increased epicardial fat has been reported in post-menopausal women (de Vos et al., 2008; Cabrera-Rego et al., 2018). The higher epicardial fat was associated with coronary artery disease and other markers of cardiovascular risk (de Vos et al., 2008; Cabrera-Rego et al., 2018). Hence the hormonal changes occurring in the menopause may also play a role in the changes within the epicardial fat.

## Potential Beneficial Effects of Drugs Targeting Epicardial Fat

Recent cardiovascular clinical trials showed that anti-diabetic medications can provide beneficial cardiovascular effects that go beyond the glucose control. Epicardial fat responds to newer pharmaceutical drugs such as glucagon like peptide one analogs (GLP-1A) (Morano et al., 2015; Dutour et al., 2016; Iacobellis et al., 2017; Iacobellis and Villasante Fricke, 2020) and sodium glucose transport 2 inhibitors (SGLT2i) (Yagi et al., 2017; Díaz-Rodríguez et al., 2018; Sato et al., 2018; Iacobellis and Gra-Menendez, 2020). A significant epicardial fat reduction has been reported in all of these studies. As both GLP-1As and SGLT2is induce weight loss, their effect on EAT is likely due to an overall fat reduction. However, the effects of GLP-1A on EAT could be directly mediated by the presence of GLP-1 receptors as recently discovered (Iacobellis et al., 2017). EAT GLP-1 receptor is associated with genes involved in fatty acid oxidation and white-to-brown fat differentiation (Yang et al., 2013; Beiroa et al., 2014; Dozio et al., 2019). GLP-1 agonistic effects o may resume EAT browning function. As brown fat activity is lost or at least significantly diminished with age, it is tempting to speculate on the potential anti-aging effects of GLP-1A. Future studies are warranted in this direction.

## Conclusions

Epicardial adipose tissue encounters changes in the transition from embryological to childhood and then to adult life. Some of these changes are physiological and simply reflecting the adaption of the tissue to different conditions and functions. However, the effect of aging on the brown fat properties of the epicardial fat seems to be the most prominent and with the greatest clinical implications. Whether the downregulation of the epicardial fat tissue is due more to aging than advancing stages of coronary artery disease, or more likely to the combination of both, would be also object of future investigations.
